# Interventions to support patients with sharing genetic test results with at-risk relatives: a synthesis without meta-analysis (SWiM)

**DOI:** 10.1038/s41431-023-01400-1

**Published:** 2023-06-21

**Authors:** Lisa Marie Ballard, Rebecca Band, Anneke M. Lucassen

**Affiliations:** 1 Clinical Ethics, Law and Society (CELS), Primary Care, Population Sciences and Medical Education, University of Southampton, Southampton General Hospital, South Academic Block, Tremona Road, Southampton, SO16 6YD UK; 2 NIHR Southampton Biomedical Research Centre, University of Southampton and University Hospital Southampton NHS Foundation Trust, Southampton, UK; 3 Health Sciences, Highfield Campus, University Road, Southampton, SO17 1BJ UK; 4 Clinical ethics, law and society (CELS), Wellcome Trust Centre for Human Genetics, University of Oxford, Oxford, OX3 7BN UK

**Keywords:** Genetic counselling, Medical ethics

## Abstract

Whilst the finding of heritable susceptibility to disease was once relatively rare, mainstreaming of genetic testing has resulted in a steady increase. Patients are often encouraged to share their genetic test results with relevant relatives, but relatives may not receive this information, leaving them without knowledge of their own risk. Therefore, strategies to help communicate such information are important. This review aimed to explore the efficacy of existing interventions to improve the sharing of genetic test results. A synthesis without meta-analysis design was used. A systematic search of Medline, CINAHL, PsychINFO, and AMED was conducted, and five studies were identified worldwide. Data were extracted for each study regarding study aim, participant characteristics, condition, intervention details, comparison, study duration, outcome measures, theory and behaviour change techniques used. Limited efficacy and application of theory was found. Knowledge, motivation and self-efficacy were not increased in any intervention. No gender differences in communication behaviour were encountered in interventions that recruited men and women. Two studies reported an evaluation of acceptability, which showed that the interventions were well received by patients and health professionals. No study reported the involvement of the target population in any phase of intervention development. Given the lack of health psychology-informed interventions in this area of clinical genetics, we recommend genetic health professionals, health psychologists and patients collaborate on all stages of future interventions that involve the cascading of genetic health information within families. We also provide guidance regarding use of theory and intervention elements for future intervention development.

## Introduction

Genetic testing is being increasingly used to identify genetic predisposition to disease due to the mainstreaming of genetic testing through, for example, the National Health Service (England) genomic medicine service. Given that these predispositions are often inherited, the results of tests in one person (the proband) can reveal risk to that person’s relatives [1, 2]. For example, finding a BRCA2 variant (associated with an increased risk of breast, ovarian, and prostate cancer) in an individual could indicate that their close relatives might also benefit from such testing. However, these relatives will only be able to decide if they would like to take up testing, and any subsequent screening or preventative measures, if they are made aware of this possibility [3].

Research into familial communication suggests that patients often believe it is their responsibility to inform relatives of any relevant genetic information [4–7] but that some would like to do so with support from their health professional (HP) [8]. Guidelines about such communication generally outline the role for HPs as one of encouraging the proband to communicate relevant information to at-risk relatives and to offer support in doing so [9]. Probands are however, left to make their own judgements on when would be the best time to pass on this information, and in what way [10]. HP assistance often includes the use of a ‘family letter’, which outlines in general terms information about the genetic finding and how a relative might seek more information [11, 12]. How – or whether – family letters are used is unclear, with clinicians often left unsure as to whether the relevant information has been shared [12].

Although patients generally understand the importance of sharing information with family members, especially those with risks of diseases that can be prevented or treated [13], they also report difficulties in doing so [14–16]. It is estimated that many relatives do not receive such information in a timely fashion [14, 17–23] meaning that some remain unaware of their potential risks for longer than necessary and some never receive this information. Studies also show that when communication does occur, it may in fact be to the wrong relatives (those not at risk) or that the information passed on is insufficient or wrong [24–28].

There are many reasons why patients do or do not inform their at-risk relatives, or delay doing so [4, 20, 28–30]. Reasons include feelings of responsibility or guilt, family dynamics, perceived recipient reactions, perceived relevance of the information and the psychological burden of coping with their own result. This psychological distress or burden caused by giving or receiving such information has been shown to be often outweighed by the health advantages of having access to screening and appropriate healthcare [6, 31, 32]. The aim of informing relatives is so that they have an opportunity to make a more informed decision they could not make without such communication. Given that a range of studies has shown that not all at-risk relatives receive such information appropriately, interventions to improve this communication are needed [4, 17]. Yet resources to facilitate this are often limited [33–35] and there is no procedure in the UK that guarantees a relative – with whom the HP usually does not have direct contact – will receive information about their genetic risks [20].

### Previous reviews in this area

Mendes et al., (2016) [34] summarised studies exploring the role of HPs in the communication of genetic health information within families and examined how such communication is addressed in clinic. The authors concluded that encouraging ‘reflective consideration’ and exploring ‘family dynamics and patterns of communication’ were helpful for HPs. Baroutsou et al., (2021) [35] reviewed interventions to facilitate familial communication of genetic health information, including interventions that focused on the gathering of a family history (i.e., [36]) and where no genetic test was conducted (i.e., [37, 38]). Zhao et al., (2022) [38] also reviewed family communication frameworks and included interventions that support the gathering of family histories. This review progresses from these broad reviews to a more detailed understanding of what interventions to facilitate communication exist, and whether, how and why they are effective. Our aims differ from previous reviews, in that they are focused on the sharing of a genetic test result; we look at the likelihood of performing the behaviour (sharing) and the quality of that behaviour (accuracy and effectiveness).

### What is needed for an intervention to be successful in changing a behaviour?

Using a theory to guide the development of an intervention will make it more likely that the intervention will be successful [39] and the need for more theory-based interventions in the area of familial communication of genetic information has been highlighted [40]. Theory in this context should explain how, when and why a behaviour change intervention does, or does not, work [41]. An essential component to an efficacious intervention is ensuring the end user is involved in every step of the development of the intervention [42]. One approach to user involvement is the ‘person-based approach’ [43]. This approach focuses on involving the people the intervention is directed at, with the aim of understanding and integrating their needs and perspectives, which increases the likelihood of uptake and intervention engagement.

### Objectives

This review aimed to explore the efficacy of existing interventions that encourage patients to share genetic health information with their relatives and what intervention elements authors identified as making the performance of the behaviour more likely. We achieved this aim through the following objectives: 1) comparing study outcomes, 2) comparing behaviour change techniques (BCTs) used, 3) describing the extent to which interventions draw on theories of behaviour change, 4) describing the extent to which the views of people from the target population were incorporated into the development of the interventions, and 5) demonstrating how barriers and facilitators can be mapped onto theory.

## Methods

A systematic review: synthesis without meta-analysis (SWiM) [44] design was used. This is an alternative method of synthesis from a meta-analysis, comprising of a narrative synthesis of effects. The studies included in this review were synthesised using this method due to methodological heterogeneity (i.e., RCTs and non-randomised and clinical diversity in relation to PICO (Population, Intervention, Comparison, Outcome)). This review follows the PRISMA Statement process [45] and the SWiM guidelines [44].

The protocol for this review can be accessed from www.crd.york.ac.uk/prospero with the registration number CRD42019121588.

### Eligibility criteria

**Fig. 1:**
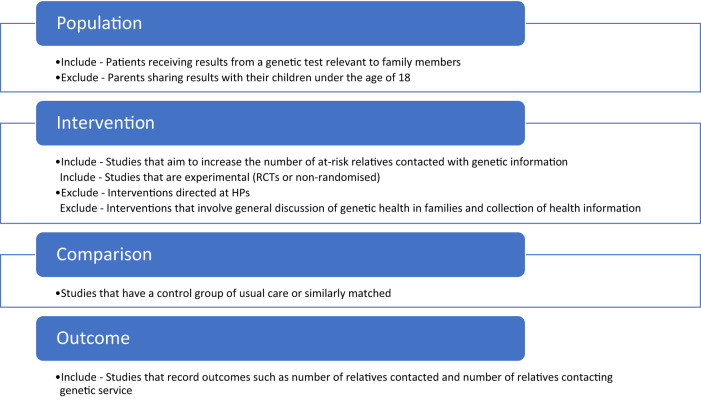
The inclusion and exclusion criteria organised into four categories. Population, Intervention, Comparison and Outcome (PICO).

PICO was used to inform the eligibility criteria (see Fig. 1). We engineered the eligibility criteria to reflect a very specific behaviour - an adult with a genetic test result sharing that result with at-risk relatives under the advice of their HP. We therefore excluded interventions aimed at collecting family histories as - although they report on familial communication - the aims and challenges of collecting a family health history are different from sharing a genetic test result. We excluded interventions aimed at supporting parents to inform children under the age of 18 as the mode and method of communicating to children of varying ages will have to be very different to those used with adults. Finally, we excluded interventions aimed at HPs because the target behaviour is different, with a unique list of barriers and facilitators.

### Information sources and search

Electronic databases Medline, CINAHL, PsychINFO, and AMED were searched in September 2019 for peer reviewed papers, the search was updated in March 2022. Grey literature searches and hand searching were performed for editorial letters, blog posts, conference proceedings and bulletins. No unpublished relevant literature was identified so no researchers/authors were contacted throughout this meta-synthesis. Supplementary papers were used, such as published protocols, to allow the coding of interventions.

Citation chaining (forward and backwards) was performed on the final articles selected for inclusion in this synthesis. After a preliminary search to refine the search terms the following terms were used: duty to inform OR family communication OR at-risk relative OR disclosure AND genetics OR genetic coun* OR genetic testing AND intervention OR randomi*ed controlled trial. Studies were selected based on the PICO characteristics in Fig. 1.

### Study selection

Abstracts were screened independently by LMB and JF using the inclusion and exclusion criteria outlined in Fig. 1. Disagreements were discussed and resolved in all cases without the need for external involvement. The updated search was conducted by LMB based on the protocol and discussions from the original search.

### Data collection process

Overall quality assessment and risk of bias was conducted using the EPHPP model (see Supplementary information) by two independent reviewers [46]. This tool leads to a rating of weak, moderate or strong based on an assessment of six categories. Data was extracted using the ‘Cochrane Data collection form for intervention reviews: RCTs and non-RCTs’. Data extracted included type of study, participants, type of intervention, theoretical basis and sections were added to the data extraction sheet for behaviour change techniques (BCTs) and evidence of researchers working with the target population in developing the intervention. BCTs were coded according to the behaviour change technique taxonomy (v1) of 93 hierarchically clustered techniques [47, 48].

### Data analysis

We synthesised the data from each study by describing study characteristics of interest, theory and target group involvement. BCTs were coded by identifying all elements of each intervention and coding them according to the BCT taxonomy (V1). Once BCTs were identified for each study, we used the Kok et al., (2016) [48] intervention mapping tables, in which they have matched relevant theory with each BCT, to highlight suitable theories. Our synthesis aimed to provide rich descriptions about mediating factors, identification of similarities and differences across studies to inform the development of theory, and identification of what has worked and has not for whom and in what circumstances. We present the findings of this synthesis themed by patient outcomes.

## Results

### Study selection

**Fig. 2:**
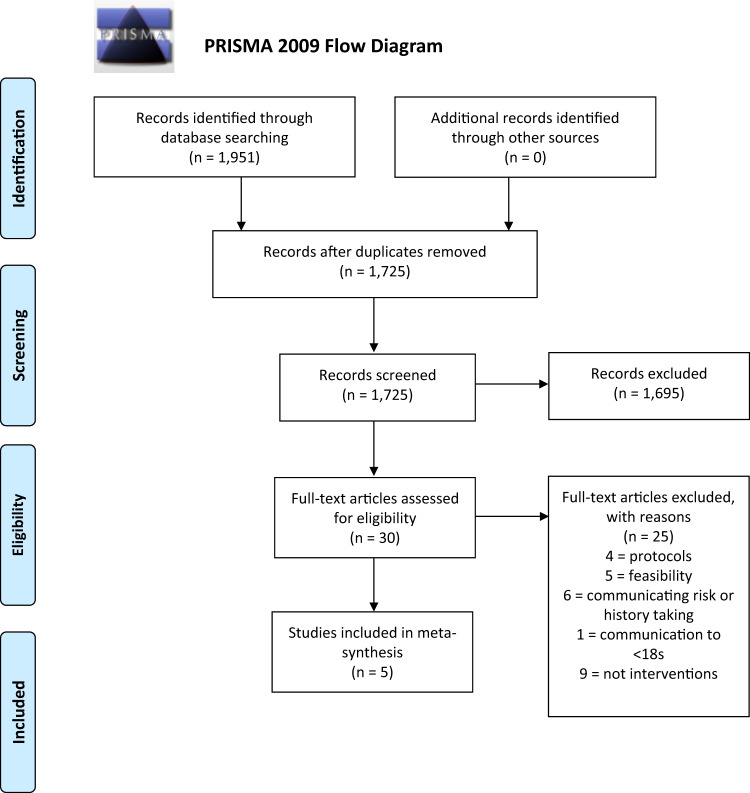
The PRISMA flow diagram illustrating the stages of the systematic review starting with the original search which identified 1951 articles and ending with the five studies included in the synthesis.

1173 papers of potential relevance were found after duplicates were removed (see Fig. 2 for PRISMA flow diagram). After scanning titles and abstracts 1149 papers were removed. From the remaining 24 full text articles 19 were excluded.

### Study characteristics

Table 1 summarises the study design and main findings for each study. The included papers were quantitative in design and were published between 2008 and 2018. The studies took place in different countries; two in Australia, two in the United States of America (USA) and one in the Netherlands. Studies looked at high risk single gene/mendelian disorders, where there were clear familial risks. They did not include, for example, moderate risk genes or polygenic scores where the risk to communicate to family members would be much less clear/certain. Three focused on heritable cancer syndromes and two looked more broadly at genetic conditions that had an implication for family members. Four studies used a randomised controlled trial design, and one used a controlled before and after study. Two studies (Eijzenga et al. 2018 [49] and Montgomery et al. 2013 [21]) included the communication of both positive and negative results (when a proband did have a variation and when they did not), whilst the remaining studies focused on the communication of results that indicated increased risk only. Overall study quality (see supplementary information) was assessed as being weak.

**Table 1:** Summary of study characteristics.

Study	Design	Sample characteristics	Intervention description	Duration of intervention	Comparison	Outcomes measured	Key findings
Forrest et al. (2008), Tasmania, Australia.	Controlled Before and After Study (CBA).	Patients with a genetic test result, showing that they carried a gene mutation. **Intervention sample** 11 probands and 76 at-risk relatives (55% women) (average 7 relatives per participant). **Control sample** 8 probands and 55 at-risk relatives (48% women) (average 7 relatives per participant). **Genetic conditions** Balanced reciprocal chromosomal translocation, hereditary breast and ovarian cancer (BRCA1/2), hereditary nonpolyposis colorectal cancer [Lynch syndrome], multiple endocrine neoplasia type 1, Peutz-Jegher syndrome, or an X-linked condition with re-productive implications.	Telephone follow-up at 2-4 weeks and 3-6 months to support communication to relatives.	Two years	Retrospective audit of files.	Proportion of at-risk relatives who had made contact with the clinical genetics service within 2 years of the diagnosis in the proband.	The difference in the frequency that at-risk relatives were “definitely informed” between the intervention and control cohorts was statistically significant (χ­^2^ 6.52, P 0.01). **Powered to identify differences** Authors did not report a power calculation and due to small sample size, it is assumed the study was not sufficiently powered.
Kardashian et al. (2012), California, USA	RCT Pilot.	Women who received genetic counselling and tested positive for a germline BRCA1/2 mutation. **Genetic conditions in sample** BRCA1/2	An educational tool in the form of a binder given to patients to take away	Between two and ten months after results disclosure.	Usual care	Knowledge. Participants’ report of sharing BRCA results with eligible family members and 2) participants’ report of relatives receiving BRCA testing.	**Intervention** First-Degree Relatives 90% (p 1.00) Second-Degree Relatives 75% (p 0.32) Cousins 63% (p 0.86) **Control** First-Degree Relatives 88% Second-Degree Relatives 38% Cousins 40% **Power to identify differences** The study was not sufficiently powered to detect differences between groups.
Montgomery et al. (2013), Philadelphia, USA.	RCT.	Women aged 18 years and older, who completed genetic testing for BRCA1/BRCA2, and who had one or more living adult FDRs with whom she would share results. **Intervention** 187 probands (100% women) **Control** 158 probands (100% women) 1385 relatives in total (51% women) (average of 4 relatives per participant) **Genetic conditions in sample** BRCA1/2	A six-step communication strategy delivered in the pre and post results counselling sessions with a resource guide given to each patient.	Three months after results disclosure.	Wellness session	Self-reported informing relatives.	99% of probands shared their genetic test results with at least one relative, and 53% shared test results with all of their FDRs. There was no significant difference between the intervention and control group in the percentage of probands sharing test results, or in their difficulty and distress in sharing test results. **Power to identify differences** The study was sufficiently powered to detect differences, with a sample of 110 needed for each condition, which was achieved.
Hodgson et al. (2016), Victoria, Australia.	RCT	Patients first in family to be diagnosed, or have a child diagnosed, with a genetic condition or as a carrier of a genetic condition that has implications for others. **Intervention** 45 probands and 554 at-risk relatives (87% women) (average of 12 relatives per participant) **Control** 50 probands and 536 at-risk relatives (88% women) (average of 11 relatives per participant) **Genetic conditions in sample:** BRCA1, BRCA2 and Lynch Syndrome, inherited cardiac conditions. A carrier for chromosomal anomalies i.e., translocations, X-linked conditions such as Fragile X syndrome and Duchenne/Becker muscular dystrophy.	Telephone follow-up at 3, 6, and 12 months based on the reciprocal engagement model.	18 months	Usual care	Proportion of at-risk relatives who contacted genetics services for information and/or genetic testing.	The adjusted OR, considering the clustering effect within families, was 1.30; and the 95% CI was 0.70–2.42, P = 0.40, so no significant difference was found between the intervention and control conditions. **Power to identify differences** The study was sufficiently powered, needing a sample of 151 relatives, which they achieved.
Eijzenga et al. (2018), Netherlands	RCT	Patients with a conclusive (pathogenic mutation) and inconclusive (yet an increased risk based on family history) DNA test result. **Intervention** 148 probands (75% women) 636 at risk relatives (no gender information reported) (average of 4.3 relatives per participant) **Control** 157 probands (75% women) 628 at risk relatives (no gender information reported) (average of 4 relatives per participant). **Genetic conditions in sample** Hereditary and familial cancer: Breast/ovarian and colorectal.	One off telephone counselling session based on motivational interviewing.	Four months	Usual care	Knowledge, motivation, self-efficacy, and self-reported informing relatives.	No differences were found between the groups for knowledge of whom to inform or information to convey, for motivation, or self-efficacy. No differences were found between the intervention and control group for correctly informed relatives. Intervention = 1st degree 94 (n) 82%, 2nd 84 (n) 75%. Control = 1st degree 91 (n) 83%, 2nd 81 (n) 78%. 60% reported over informing (informing relatives that were not at increased risk). **Power to identify differences** As there was limited information regarding the outcomes, the authors based their power calculations on expected differences between groups. 132 participants were required per group, which they did achieve, therefore the study was sufficiently powered.

### Study results

Of the five interventions, three used prompting (i.e., calling to see how the proband is getting on with sharing their result with relevant relatives) as the main intervention along with problem solving support if they were struggling to share or had encountered any issues (Forrest et al., 2008 [18], Hodgson et al., 2016 [50], and Eijzenga et al., 2018). Kardashian et al. (2012) [19] used education about genetic findings and their relevance for relatives as the intervention and Montgomery et al. (2013) focused on building skills in communication of genetic test results, such as who, what and how to tell as well as identifying barriers to sharing. Three main outcomes were identified as being used by the studies in this review: 1) participant self-report of informing relatives (used in three studies (Kardashian et al., 2012; Montgomery et al., 2013; Eijzenga et al., 2018)); 2) relatives contacting genetics services (used in two studies (Forrest et al., 2008, and Hodgson et al., 2016)); and 3) relatives accessing testing (used in two studies (Forrest et al., 2008 and Kardashian et al., 2012)). One study found the intervention to be effective. There were also six additional outcome measures identified. Table 2 shows how the groupings were developed; each grouping is described in more detail below.

**Table 2:** Development of outcome groupings.

Outcome measure	Forrest et al. (2008)	Kardashian et al. (2012)	Montgomery et al. (2013)	Hodgson et al. (2016)	Eijzenga et al. (2018)
**Primary outcome measures**					
Self-report		**x**	**x**		**x**
Relative contacting service	**x**			**x**	
Relative tested	**x**	**x**			
**Secondary outcome measures**					
Knowledge		**x**			**x**
Motivation					**x**
Self-efficacy					**x**
Distress			**x**		
Gender differences	**x**	**x**^a^	**x**		**x**
Evaluation (feasibility/ acceptability)		**x**			**x**

^a^Reported on gender differences even though no data on relative’s gender was reported.

#### Self-report

Of the three studies that used self-reported sharing of information with relatives (Kardashian et al., 2012; Montgomery et al., 2013; Eijzenga et al., 2018) none found a significant difference between the intervention and control groups. Kardashian et al., (2012) and Montgomery et al., (2013) did show an effect in the direction of the intervention, however Eijzenga et al., (2018) found the direction of effect to be towards the control condition (see Table 3 for direction of effect).

**Table 3:** Direction of effect for primary outcome measures.

Studies	Sample Size	Control (%)	Intervention (%)	Percentage increase or decrease between control and intervention group
Forrest et al. (2008)	Control: 8 probands and 55 at-risk relatives. Intervention: 11 probands and 76 at-risk relatives.	36 relatives contacted 33 relatives tested	61 relatives contacted 57 relatives tested	69% increase 73% increase
Kardashian et al. (2012)	Control: 10 probands and 134 at-risk relatives. Intervention: 9 probands and 73 at-risk relatives.	Self-reported sharing with: 88 FDRs 38 SDRs Relatives accessing testing: 25 FDRs 67 SDRs	Self-reported sharing with: 90 FDRs 75 SDRs Relatives accessing testing: 20 FRDs 14 SDRs	2% increase 97% increase 20% decrease 79% decrease
Montgomery et al. (2013)	Control: 158 probands. 1385 relatives in total. Intervention: 187 probands.	One relative informed 99 All relatives informed 53	One relative informed 99 All relatives informed 54	0% increase 2% increase
Hodgson et al. (2016)	Control: 50 probands and 536 at-risk relatives. Intervention: 45 probands and 554 at-risk relatives.	Relatives accessing testing 21	Relatives accessing testing 26	24% increase
Eijzenga et al. (2018)	Control: 157 probands and 628 at risk relatives. Intervention: 148 probands and 636 at risk relatives.	83 FDRs correctly informed 78 SDRs correctly informed	82 FRDs correctly informed 75 SDRs correctly informed	1% decrease 4% decrease

*FDR* first degree relative, *SDR* second degree relative.

#### Whether or not relatives contacted the genetic service

Two interventions used relatives of probands contacting the genetics services as an outcome measure (Forrest et al., 2008 and Hodgson et al., 2016). Forrest et al., (2008) was the only study where significantly more relatives of participants in the intervention group were informed of their risk and sought support from the genetic centre compared to the control group (χ^2^ = 6.52, P 0.01). However, there was no mention of a power calculation to determine sample size; the only acknowledgement of sample size was stating that the number of families in the study was ‘relatively small’. Hodgson et al.’s (2016) did not find a significant difference between the intervention and the control condition for proportion of relatives contacting the genetics service, but the direction of effect was towards the intervention group.

#### Relatives tested

Two studies measured whether relatives had taken up testing. Significantly more relatives underwent testing in the intervention group in the Forrest et al. (2008) study. Kardashian et al. (2012) did not find a significant difference and the direction of effect was negative (see Table 3).

#### Knowledge

Two studies used knowledge as an outcome measure; neither found that the intervention increase knowledge regarding whom to inform and what information to share (Kardashian et al. 2012 & Eijzenga et al., 2018).

#### Motivation, self-efficacy and distress

No intervention significantly increased motivation and self-efficacy (Eijzenga et al., 2018) and Montgomery et al., (2013) found no increase in distress.

#### Gender differences

Both Kardashian et al., (2012) and Montgomery et al., (2013) limited their studies to the recruitment of women. Other studies found that women are more likely to communicate genetic information to at-risk relatives than men [51], which may explain why the interventions were not found to be effective. Forrest et al., (2012) and Eijzenga et al., (2018) recruited both men and women probands, but found no gender differences. Moreover, there were differences reported for gender of relatives. For example, Forrest et al., (2008), Kardashian et al., (2012) and Montgomery et al., (2013) found that female at-risk relatives were more likely to be informed than male at-risk relatives. However, none of the studies were sufficiently powered for subgroup analysis.

#### Evaluation

Two studies reported feedback from HPs delivering the intervention and/or participants receiving it. Kardasian et al., (2012) found that even though the introduction of an educational tool in the form of a binder given to patients added 30 minutes to genetic counsellors’ workload, they were still positive about its use as it helped them structure their consultations and it was well received by patients. The intervention in the Eijzenga et al., (2018) study was found to be acceptable to patients; 96% of participants found the telephone counselling to be useful and 96% did not find the intervention to be confrontational.

### Additional data synthesis based on review objectives

#### Involvement of the target group in intervention development

None of the studies involved the target population in the development of the intervention (see Table 2). Two studies, Kardashian et al., (2012) and Montgomery et al., (2013), consulted the HPs who would be delivering the intervention.

#### Behaviour change techniques (BCT) coded from each intervention

We coded each intervention to determine which BCTs were implemented using a published and widely used taxonomy (v1) (Michie et al., 2013) (tables with BCTs for each study are in supplementary information). De Vasconcelos et al., (2018) showed that effective interventions have a median number of nine BCTs (range 3-25). Our review found that the maximum number of techniques used was seven. The Forrest et al. (2008) intervention was clearly explained, making coding of techniques easier, and used the most BCTs (seven). Eijzenga et al.’s (2018) intervention was well described making it easier to identify the five BCTs used. Hodgson et al. (2016), Montgomery et al. (2013) and Kardashian et al. (2012) did not describe the intervention in sufficient detail, making the identification of BCTs difficult. From the details provided, we identified that the Hodgson et al. (2016) intervention included six BCTs and Montgomery et al. (2013) and Kardashian et al. (2012) four. Kardashian et al. (2012) also did not justify any of the intervention components, or a link between the target behaviour and the intervention.

#### Use of behaviour change theory for intervention development

Once the BCTs were coded, we used the Kok et al., (2016) intervention mapping tables, in which relevant theory is matched with each BCT, to highlight suitable theories (see supplementary information). From their ‘methods to change awareness and risk perception’ table, Kok et al. (2016) summarise that the Health Belief Model, Precaution-Adoption Process Model, Trans-Theoretical Model (Stages of Change) as potential theories to facilitate providing information regarding the causes and consequences of a behaviour being performed (or not). The Precaution-Adoption Model also facilitates functions such as cost/benefit analysis of action and inaction and constructing images of future gains and losses. Framing – from the Protection Motivation Theory - may also be a useful concept for the sharing of genetic health information, whereby messages from HPs are gain-framed (the advantages of performing the behaviour) and loss-framed (the disadvantages of not performing the behaviour). Because the target behaviour here is one that affects another person (and often more than one person) theories that facilitate the shifting of perspectives, such as Theories of Stigma and Discrimination, could help the proband to take on the perspective of their relative. Similarly, so could Theory of Planned Behaviour; Reasoned Action Approach; Social Comparison Theory, all of which have elements of considering others’ approval or disapproval.

Montgomery et al., (2013) was the only study reporting the use of theory. They used the Theory of Planned Behaviour (TPB) and measured components such as, attitudes, subjective norms, perceived behavioural control. They showed that three TPB variables predicted intention to inform, though in a further analysis they found that attitude did not predict actual sharing, whereas social norms and perceived control did. The social norms and perceived control variables were strong predictors of behaviour. However, there was no clear explanation as to how the theoretical components related to the intervention. Hodgson et al., (2016) did describe the development of the intervention being informed by the Reciprocal Engagement Model of genetic counselling, however, we did not classify this as a behaviour change theory because it described standard practice. In addition, Eijzenga et al., (2018) stated that their intervention was based on Motivational Interviewing. We did not classify this as a behaviour change theory as it is more of a ‘counselling style’ and it does not explain how the intervention leads to change.

### Moving this field of enquiry forward

**Fig. 3:**
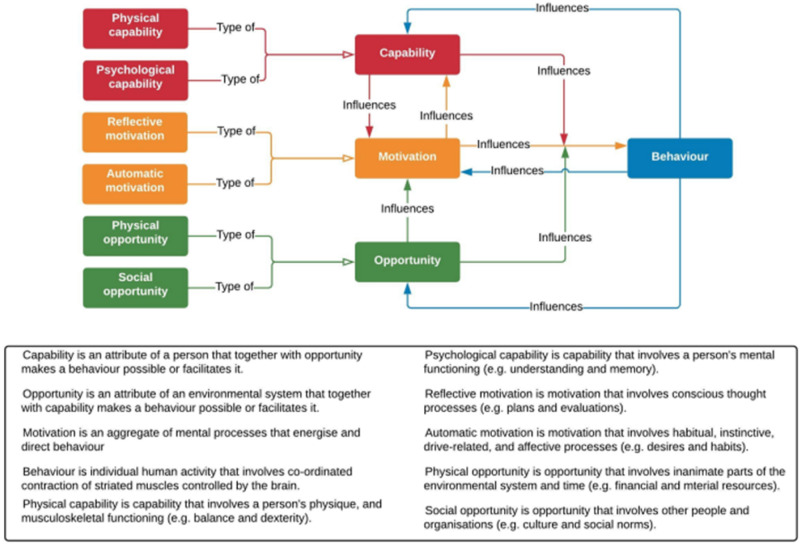
The COM-B model which theorises that Capability, Opportunity, and Motivation lead to Behaviour. The three key concepts are divided into two further categories to capture distinct elements of each concept [61].

**Fig. 4:**
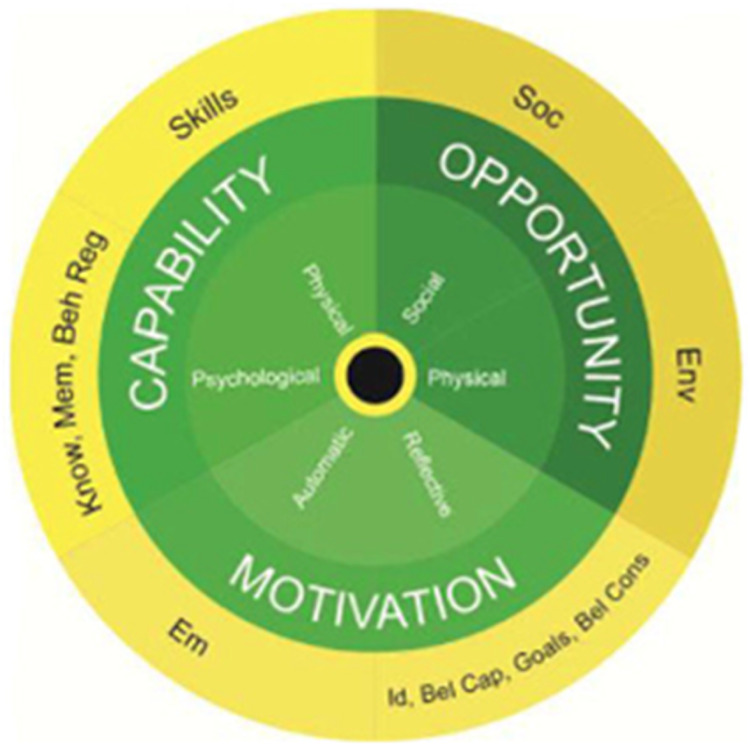
The Theoretic Domains Framework includes the COM-B model. It is broken down into 14 domains to enhance specificity [62].

Given the lack of intervention development and use of theory in this area we have completed an additional step to this review by demonstrating how to develop a theory-informed intervention, using the COM-B model (see Fig. 3), which theorises that capability (C), opportunity (O) and motivation (M) need to be present for a behaviour (B) to happen. There are many behaviour change theories as listed above, but many are complex making them inaccessible to many researchers and HPs [52]. Michie and colleagues developed the Behaviour Change Wheel (COM-B and the Theoretical Domains Framework) to address some of these challenges (Ibid). The COM-B model can be broken down further into 14 domains, which are organised under the Theoretical Domains Framework (see Fig. 4) [53, 54]. We used the four steps outlined in French et al., (2012) to demonstrate how to apply a behaviour change model to the patient behaviour of sharing genetic health information with relatives indicated by their HP, which can been seen in Table 4. Here we conducted a behavioural analysis based on previous literature (separate from the coding of BCTs in the interventions included in this review) and then mapped the BCTs onto the COM-B model. The left column of Table 4 details barriers and facilitators identified from the wider literature, presented in sections corresponding with the COM-B model. The right column matches behaviour change techniques to the particular barrier or facilitator. For example, if a researcher identified that only being advised once to share results with at-risk relatives reduces the likelihood that behaviour will be performed, they may wish to build prompts and cues into the intervention.

**Table 4:** Demonstration of how the COM-B Model could be applied to intervention development.

**Barriers and facilitators**	**BCTs and implications for interventions**^**a**^
COM-B: Capability	
Theoretical domain: Knowledge	
**Negative result is not as informative/useful. For example:**	**1. Goals and planning**
If proband tests negative and they do not perceive themselves to be directly affected, they are less likely to inform, as they feel they may not be taken seriously (63).	1.3 Goal setting (outcome) – share x information with y relatives
**Misconceptions about heritability and illness representations. For example:**	**4. Shaping knowledge**
Informing those that are not at risk i.e., if the proband is not a carrier, but then informing children of risk (14). Misconceptions that men are not at risk of BRCA1/2 alterations (25).	4.1 Instructions on how to perform the behaviour
**Poor comprehension of information. For example:**	**5. Natural consequences**
Do not communicate as think family are not at risk (64). Not all or correct information is communicated (24).	5.1 Information about health consequences of performing the behaviour 5.3 Information about social consequences 5.6 Information about emotional consequences
Additional notes: Include information about the importance of sharing negative results and myth busting. Consider adapting the ‘to whom it may concern letter from the HP needs to be easy to read, with infographics and less words to send as simple a message as possible to encourage relative to contact clinic, emphasise that you won’t have to have a test, just discuss the best option. Consider the use of web applications for intervention and sharing of results (Anonymous et al., 2019a).	
COM-B: Capability	
Theoretical domain: Skills	
**Competence, ability, practice, interpersonal skills, coping strategies. For example:**	**1. Goals and planning** **6. Comparison of the behaviour**
If the first attempt fails, it becomes much less likely the information will be communicated (65). Do not know what to say (65). Men less likely to communicate than women (66).	1.4 Action planning – how, when, who, where. Preparation – start with the easiest person first, use that experience to help with relatives they think will be more difficult. 6.1 Demonstration of the behaviour 6.2 Social comparison
Additional notes: Consider using scripts and templates for emails and message to help with action planning.	
COM-B: Capability	
Theoretical domain: Memory, attention and decision-making	
**Attention. For example:**	**7. Associations**
The proband only being asked to share results with relatives only once at a counselling appointment.	7.1 Prompts/cues
COM-B: Capability.	
Theoretical domain: Behavioural regulation	
No follow up from health professional regarding whether the proband has communicated their test result to relevant others.	**2. Feedback and monitoring** 2.4 Self-monitoring of outcome(s) of behaviour 2.5 Monitoring outcome(s) of behaviour by others without feedback 2.7 Feedback on outcome(s) of behaviour
COM-B: Opportunity	
Theoretical domain: Social influences.	
**Barriers. For example:**	**3. Social support**
Family communication patterns (other family members normally do this communication) (51).	3.1 Social support (unspecified)
Estrangement and family disruption (67).	3.3 Social support (emotional)
**Facilitators. For example:**	
Physical and emotional closeness, family cohesion, open communication, good relationship, in close contact (51,63,64,66,68).	Consider using modelling – construct vignettes based on real life case studies, emphasising informing relevant relatives that probands are also not close to. Digital assistance to enable quick and anonymous communication if necessary (Anonymous et al., 2019a). Consider adding an application which maps closeness of each person who needs informing, so more attention can be directed to the those less likely to be contacted.
COM-B: Motivation	
Theoretical domain: Emotion	
**Barriers. For example:**	**5. Natural consequences**
Proband has felt anxious for years about risk and does not want relatives to suffer the same worry (30,64).	5.5 Anticipated regret – induce or raise awareness of implications of not performing the behaviour
Proband felt burden, guilt and anxiety of passing on the risk (25).	5.6 Information about emotional consequences
Emotionally difficult to share the information, guilty, anxious, poor psychological functioning associated with greater perceived barriers (14).	**11. Regulation** 11.2 Reduce negative emotions/increase positive emotions
**Facilitators. For example:**	
Wish to prevent disease and anticipated regret by not sharing (14,63).	11.2 Reduce negative emotions/increase positive emotions
COM-B: Motivation	
Theoretical domain: Social role and identity	
**Social and group norms, boundaries and roles. For example:**	**13. Identity**
Family communication patterns (e.g., other family members normally do this communication) (51).	13.1 Identification as self as role model 13.3 Incompatible beliefs – draw attention to discrepancies 13.4 Valued self-identity – identify cherished values to confirm identity 13.5 Identity associated with changed behaviour – person who communicates health information to relatives
COM-B: Motivation	
Theoretical domain: Beliefs about capability	
**Self-efficacy, perceived competence and empowerment. For example:**	**15. Self-belief**
Encouragement and support from HPs (14).	15.2 Mental rehearsal of successful performance 15.3 Focus on past success
COM-B: Motivation	
Theoretical domain: Goals	
**Barriers. For example:**	**1. Goals and planning**
Not being able to find the right time to communicate results (26).	1.5 Review behaviour goal(s) – review outcome and make modifications accordingly
Deciding to communicate at a time when it is more actionable i.e., prostate cancer when in 40 s (25).	1.6 Discrepancy between current behaviour and goal – point out that not all relatives informed
**Facilitators. For example:**	
Intrinsic motivation: Obligation/responsibility (14).	1.7 Review outcome goal(s) 1.8 Behavioural contract – written specification of the behaviour witnessed by another 1.9 Commitment – ask the person to use an “I will” statement to affirm commitment to behaviour **9. Comparison of outcomes** 9.1 Credible source – communication from a credible source for or against the behaviour 9.2 Pros and cons - of doing and not doing the behaviour 9.3 Comparative imagining of future outcomes – of doing and not doing the behaviour
COM-B: Motivation	
Theoretical domain: Beliefs about consequences	
**Assumptions made about relatives wishes/state. For example:**	**5. Natural consequences**
The perception that the relative lacks sufficient maturity (70).	5.5 Anticipated regret – induce or raise awareness of implications of not performing the behaviour
Assessing relative’s vulnerability or receptivity, didn’t want to add to other burdens (25,26,68). Relative had not indicated a readiness to know (24). Proband considerers relatives right not to know unpleasant or unwanted information (25). Believes that relatives would not be interested in information (66). Fear of getting cancer would prevent their relative from pursing cancer risk information (68).	5.6 Information about emotional consequences

Additional notes: Right not to know – do we really know someone well enough to decide if they would like to know or not? Use vignettes to highlight ethical dilemmas.

^a^The number next to each technique refers to the corresponding number in the Behaviour Change Taxonomy v1 (Michie et al. 2013).

## Discussion

Our review explored interventions designed to increase the likelihood that probands shared relevant genetic health information with their appropriate relatives. We identified five such studies. Each had identified some weaknesses to their approach, and we highlighted further ones. We recognise that the behaviour of sharing genetic test results with relatives is a difficult behaviour to firstly influence and then to measure the effectiveness of that influence. It will always be difficult to measure communication among people who are not in direct contact with the health system, furthermore, the urgency to do so will vary considerably depending on the condition and the ages and relatedness of family members. Some relatives may have been well informed but chosen not to pursue genetic testing. Arguably, at least in some cases the intervention will have been helpful, but relatives made informed decisions not to seek a referral.

The Forrest et al., (2008) intervention, was the only one to find a significant difference in favour of the intervention. However, the sample was small and no power calculation was reported, meaning the significance of this is uncertain [55]. In addition, the authors highlighted that the geographical features of the clinic meant there is typically little migration and that this may influence the generalisability of the intervention. The intervention was relatively simple, indicating that prompting could work as an intervention element. No significant difference was found in the studies using relatives undergoing testing as the primary outcome measure, however one study found more relatives in the intervention condition underwent testing. Synthesising the secondary outcomes found that knowledge, motivation and self-efficacy were not increased by any intervention, but neither were negative consequences such as distress. No gender differences were observed in interventions that recruited both men and women. Regarding evaluation of interventions, none reported the involvement of the target population in any phase of intervention development. Two studies reported a basic evaluation, indicating the interventions were well received by patients and HPs.

We found limited application of theory and very few BCTs in intervention descriptions. When designing interventions to make it more or less likely a behaviour is performed, many interventions are designed using the ‘ISLAGIATT’ principle [56]. This principle, coined by Martin Eccles, stands for “It seemed like a good idea at the time”. ISLAGIATT reflects an implicit common-sense approach consisting of personal experience and a brief analysis of the behaviour. For an evidence-based approach, a thorough behavioural analysis and a theory or model should be used to inform the intervention content or “active ingredients” [53]. Theory in this context should explain how, when and why a behaviour change intervention does, or does not, work [41]. Using a theory to guide the development of an intervention will make it more likely that the intervention will be successful [39]. The systematic application of theory means that theory should inform the design; the theoretical constructs should guide the choice of intervention components, and the evaluation of the intervention (Ibid).

Only one study (Montgomery et al., 2013) used a theory of behaviour change to inform the intervention functions. The authors found that social norms, such as perceiving relatives to be supportive of testing, are a strong predictor of behaviour. Perceived control was another predictor, potentially indicating proband confidence in communicating with relatives may lead to high rates of sharing. In this study, BCTs were minimal and there was no clear justification for the intervention components. Some intervention descriptions were too brief to be able to confidently identify the BCTs used. Given the minimal use of theory, the limited reference to health psychology, behaviour change and implementation science literature and the large body of literature detailing the barriers and facilitators to sharing genetic health information with relatives we have applied a theoretical framework to this behaviour, described in detail above and summarised in Table 4.

### Recommendations

- *Interventions in clinic are acceptable and feasible* - accounts were positive from those delivering and those receiving the interventions, indicating promise that it is feasible and acceptable to implement interventions into the clinical setting.
- *Simple solutions to avoid psychological overburdening* - Eijzenga et al., (2018) found that 60% of participants reported informing relatives who were not at increased risk, as patients were often unaware of the exact message they were meant to be communicating, leading to a ‘better safe than sorry’ approach. Informing more relatives than necessary is overburdening the patient and their relatives. Interventions that make it very clear which relatives to inform and what information to share are needed [28]. One such example is myKinMatters [57], a web application which supports the proband to create a family tree, have their clinician indicate on the tree who to contact, upload their test results and electronically send them to the indicated relatives.
- *Digital interventions should be explored* – none of the interventions reviewed had a digital element, although they were situated within genetics clinics and in addition, not particularly current. Digital behaviour change interventions are increasingly being used in healthcare as they have evidence-based potential to improve health, can be easily tailored, are generally cheaper than HP-delivered interventions and can be rolled out at scale without much human resource [58].
- *Target interventions* - two interventions recruited women participants exclusively, and also reported high amounts of communication in both control and intervention conditions, indicating that future interventions could be targeted towards groups of patients who are less likely to share genetic health information with relatives i.e., men.
- *Involve the end user in all elements of intervention development* – studies in the review contained very little involvement of the target group in intervention development. It is essential that the target user is involved in the whole intervention development process, this will increase the likelihood of adoption, engagement and health outcomes [42]. Guidance regarding the previously mentioned Person-Based Approach can be found here: https://www.personbasedapproach.org/ which contains information, tutorials and resources.

### Future research

Future development of interventions in this area should consider the use of behaviour change theories and models. Genetics HPs could work alongside behavioural scientists to gain support regarding the use of behaviour change theory in intervention development [59]. Behaviour change research has been successfully applied to other areas of clinical genetic practice, such as, the study of personalised genetic risk information and health behaviour change [60]. The interventions in this review did not significantly increase *Motivation* or *Capability* (self-efficacy), however, we have demonstrated how the COM-B model can be used to develop interventions and what BCTs would be most helpful. Exploring the notion of responsibility to inform is also required to understand HP and patient roles, perspectives and assumptions. Moreover, a professional and social duty to inform at-risk relatives exists, with the legal and ethical aspects of both being qualitatively different. Comparing the practice of cascading health information in other areas of healthcare, such as infectious disease and sexual health, may also evolve thinking in this area. In addition, we must acknowledge that theories beyond behaviour change, and implementation science may be relevant and require exploration given the multilevel barriers within cascade screening in the clinical setting.

### Limitations

When examining the primary outcome measures, we found issues with each. Self-report could have an interventional function (i.e., participants are more likely to inform relatives if prompted) and also suffer from response bias (the tendency for a participant to falsely report the outcome they think the researcher wants to hear). Relatives contacting the genetic service and relatives being tested as outcome measures are problematic as this approach misses those relatives who were told but who made an informed decision not to be tested, as well as those relatives that were referred to a geographically different genetic service. However, it could be argued that the ‘gold standard’ is that every relative would have a counselling session with the genetic service. This highlights that interventions may need to target other outcomes in addition to a numerical count of relatives informed i.e., capability, opportunity and motivation [41]. And importantly – different outcomes (i.e., information sharing verses relatives contacting the service) will likely have different target behaviours.

There are several limitations at a review-level. Firstly, we included only studies published in English, due to resource constraints, meaning that articles could have been missed. Secondly, due to the heterogenous nature of the outcome measures used we were unable to conduct a meta-analysis, therefore we conducted a narrative synthesis of interventions.

## Conclusion

It is surprising that in the rapidly expanding field of genetics, which receives so much political attention, that very few studies – and not one in the UK - have paid attention to supporting probands in the communication of relevant findings to relatives. However, it is less surprising that those developing these interventions have little experience of behaviour change or implementation science theory and practice due to disciplinary silos. Health psychology has a lot to offer this relatively untapped area of medicine and we recommend health psychologists; genetics HPs and their patients work together to make the cascading of genetic health information more effective in reducing morbidity and mortality. This review has highlighted factors of importance, which we hope will focus attention on this much neglected area.

## Supplementary information

Supplementary Information

## Data Availability

The data that support the findings of this study are available from the corresponding author upon reasonable request.
