# Supplementary material for: Interventions to support patients with sharing genetic test results with at-risk relatives: a synthesis without meta-analysis (SWiM)
Source: Eur J Hum Genet. 2023 Jun 21;31(9):988–1002. doi: 10.1038/s41431-023-01400-1 (PMC10474271; doi:10.1038/s41431-023-01400-1)
Supplement: Supplementary file 1 — Supplementary Information [file 41431_2023_1400_MOESM1_ESM.pdf]

SUPPLEMENTARY INFORMATION

Supplementary A. Quality assessment and risk of bias rating of the studies using the EPHPP model, performed by two independent reviewers.

Overall quality assessment and risk of bias was conducted using the EPHPP model by two independent reviewers (1). This tool leads to a rating of weak, moderate or strong based on an assessment of six categories. Data was extracted using the 'Cochrane Data collection form for intervention reviews: RCTs and non-RCTs'.

**Study**	**Forrest et al (2008)**	**Kardashian et al (2012)**	**Montgomery et al (2013)**	**Hodgson et al (2016)**	**Eijzenga et al 2018**
**Selection Bias**	Moderate	Weak	Moderate	Weak	Moderate
**Study design**	Weak	Moderate	Strong	Strong	Strong
**Confounders**	Weak	Strong	Strong	Weak	Strong
**Blinding**	Moderate	Moderate	Moderate	Moderate	Moderate
**Data collection method**	Weak	Moderate	Weak	Weak	Weak
**Withdrawal & dropout**	Moderate	Weak	Weak	Weak	Moderate
**Overall rating**	Weak	Weak	Weak	Weak	Moderate

Supplementary B. Behaviour change techniques used (only BCT in addition to those used in the control/usual care are reported) and appropriate theory for each reviewed study.

We coded each intervention to determine which BCTs were used using a published and widely used taxonomy (v1) (2). De Vasconcelos et al., (2018) showed that effective interventions have a median number of nine BCTs (range 3-25). Our review found that the maximum number of techniques used was seven. The Forrest et al. (2008) intervention was clearly explained, making coding of techniques easier, and used the most BCTs (seven). Eijzenga et al.'s (2018) intervention was well described making it easier to identify the five BCTs used. Hodgson et al. (2016), Montgomery et al. (2013) and Kardashian et al. (2012) did not describe the intervention in sufficient detail, making the identification of BCTs difficult. From the details provided, we identified that the Hodgson et al. (2016) intervention included six BCTs and Montgomery et al. (2013) and Kardashian et al. (2012) four. Kardashian et al. (2012) also

did not justify any of the intervention components, or a link between the target behaviour and the intervention. Once the BCTs were coded, we used the Kok et al., (2016) intervention mapping tables, in which relevant theory is matched with each BCT, to highlight suitable theories. From their 'methods to change awareness and risk perception' table, Kok et al. (2016) summarise that the Health Belief Model, Precaution-Adoption Process Model, Trans-Theoretical Model (Stages of Change) as potential theories to facilitate providing information regarding the causes and consequences of a behaviour being performed (or not). Each study in the review has a separate table below.

Behaviour change techniques and relevant theory for Forrest et al. (2008)

Time point	Intervention component	Behaviour change technique from V1 taxonomy	Relevant theory from Kok (2016) paper
Before result disclosure	Pedigree reviewed and at-risk relatives identified for information disclosure.	1.3 Goal setting (outcome)<br>Set or agree on a goal defined in terms of a positive outcome of wanted behaviour.	Diffusion of Innovations Theory, Theories of Power, Models of Community Organisation, Self-Determination Theory, Goal-Setting Theory, Theories of Self-Regulation
At result disclosure	Specific discussion in consultation using pedigree to identify at-risk relatives	1.3 Goal setting (outcome)<br>Set or agree on a goal defined in terms of a positive outcome of wanted behaviour.	Same as above
At result disclosure	Facilitate discussion about importance of disclosure to at-risk relatives	5.1 Information about health consequences<br>Provide information (e.g., written, verbal, visual) about health consequences of	Theory of Planned Behaviour, Reasoned Action Approach, Theories of Learning, Social Cognitive Theory

		performing the behaviour.	
At result disclosure	Follow-up letter also documented the importance of disclosure to at-risk relatives.	5.1 Information about health consequences<br>Provide information (e.g., written, verbal, visual) about health consequences of performing the behaviour.	Same as above
2–4 weeks post result disclosure	Specific discussion and documentation which at-risk relatives have been informed	1.5 Review behaviour goal(s)<br>Review behaviour goal(s) jointly with the person and consider modifying goal(s) or behaviour change strategy in light of achievement. This may lead to re-setting the same goal, a small change in that goal or setting a new goal instead of (or in addition to) the first, or no change.	Self-Determination Theory, Theories of Self-Regulation
2–4 weeks post result disclosure	If at-risk relatives have not been informed, further exploration/ counselling and offer of guidance about how to approach relatives.	1.2 Problem solving<br>Analyse, or prompt the person to analyse, factors influencing the behaviour and generate or select strategies that include overcoming barriers and/or increasing facilitators.	Self-Determination Theory, Theories of Self-Regulation, Social Cognitive Theory, Attribution Theory; Theories of Goal Directed Behaviour.
3–6 months post result disclosure (via telephone)	Proband was re-contacted and asked whether at-risk relatives had now been informed (if at 2-4 weeks proband stated they had not informed relatives or at 3-6 months all target relatives had not yet made contact with the genetic service). If all targeted relatives had not been	1.5 Review behaviour goal(s)<br>Review behaviour goal(s) jointly with the person and consider modifying goal(s) or behaviour change strategy in light of achievement. This may lead to re-setting the same goal, a small change in that goal or setting a new goal instead of (or in addition to) the first, or no change.	See above

	informed, reasons were sought and documented.	2.7 Feedback on outcome(s) of behaviour<br>Monitor and provide feedback on the outcome of performance of the behaviour	Elaboration Likelihood Model; Social Cognitive Theory
3–6 months post result disclosure (via telephone)	If all targeted relatives had not been informed. Offer was made to write a letter explaining that a genetic condition was in the family and suggesting that contact be made with the genetic service for further information. This letter was either given to the proband for distribution or mailed directly to relatives according to preference of the proband.	12.5 Adding objects to the environment<br>Add objects to the environment in order to facilitate performance of the behaviour.<br><br>3.2 Social support (practical)<br>Advise on, arrange, or provide practical help (e.g. from friends, relatives, colleagues, ‘buddies’ or staff) for performance of the behaviour.	Social Cognitive Theory<br><br>Theories of Learning; Goal-Setting Theory, Social Cognitive Theory

Behaviour change techniques and relevant theory for Hodgson et al. 2016

Time point	Intervention component	Behaviour change technique from V1 taxonomy	Relevant theory from Kok (2016) paper
Baseline	At the time of their initial genetic consultation all probands receive standard care concerning family communication from the genetic counsellor. This generally involves a discussion of possible implications for other family members and the offer of explanatory letters for the proband to pass on to at-risk relatives	5.1 Information about health consequences<br>Provide information (e.g., written, verbal, visual) about health consequences of performing the behaviour.<br>12.5 Adding objects to the environment<br>Add objects to the environment in order to facilitate performance of the behaviour	Theory of Planned Behaviour, Reasoned Action Approach, Theories of Learning, Social Cognitive Theory<br><br>Social Cognitive Theory

3, 6, and 12 months	Supporting adjustment of the participant to their own genetic status	No BCT assigned	-
3, 6, and 12 months	recognising and exploring the conscious and unconscious barriers to communication	1.2 Problem solving Analyse, or prompt the person to analyse, factors influencing the behaviour and generate or select strategies that include overcoming barriers and/or increasing facilitators.	Self-Determination Theory, Theories of Self-Regulation, Social Cognitive Theory, Attribution Theory; Theories of Goal Directed Behaviour.
3, 6, and 12 months	which relatives have been informed and their responses	1.5 Review behaviour goal(s) Review behaviour goal(s) jointly with the person and consider modifying goal(s) or behaviour change strategy in light of achievement. This may lead to re-setting the same goal, a small change in that goal or setting a new goal instead of (or in addition to) the first, or no change.	Self-Determination Theory, Theories of Self-Regulation
3, 6, and 12 months	Maintaining or enhancing the participant's capacity for communication; by addressing misconceptions for example	Not enough detail to assign BCT	-
3, 6, and 12 months	Facilitating decision-making	Not enough detail to assign BCT	-
3, 6, and 12 months	Resolving ambivalence	Not enough detail to assign BCT	-
3, 6, and 12 months	Planning to act. Here the participant intends to	1.4 Action planning Prompt detailed planning of	Implementation intentions

	communicate, options are elicited, a plan developed, and potential scenarios explored to prepare the participant	performance of the behaviour (must include at least one of context, frequency, duration and intensity).	(Theories of Goal Directed Behaviour)
	'positive', 'negative' and 'neutral' reasons for not talking about genetics with relatives	9.2 Pros and cons Advise the person to identify and compare reasons for wanting (pros) and not wanting to (cons) change the behaviour	Precaution-Adoption Process Model

Behaviour change techniques and relevant theory for Kardashian et al (2012)

Time point	Intervention component	Behaviour change technique from V1 taxonomy	Relevant theory from Kok (2016) paper
Baseline	At this in-person visit, the implications of these results are discussed as they relate to the patient and to the patient's relatives. A longer personalized medical report (typically 3-4 pages) is sent to the patient by mail that describes and reviews the genetic testing process and the implications of the patient's BRCA test results. BRCA carriers are therefore informed in person and by mail of the importance of sharing genetic test results with family members. A single page "family letter" is provided to patients who express an	5.1 Information about health consequences Provide information (e.g., written, verbal, visual) about health consequences of performing the behaviour.<br><br>12.5 Adding objects to the environment Add objects to the environment in order to facilitate performance of the behaviour	Theory of Planned Behaviour, Reasoned Action Approach, Theories of Learning, Social Cognitive Theory<br><br>Social Cognitive Theory

	interest in receiving help with communicating their BRCA results to relatives		
Binder given to the patient at the in-person results disclosure visit	Personalized recommendations for surveillance and prevention	Not a behaviour change technique related to the behaviour of informing relative of their risk	-
	Fact sheet addressing frequently asked questions regarding cancer risk, costs of genetic testing, and insurance issues regarding genetic testing	Not a behaviour change technique related to the behaviour of informing relative of their risk	-
	Contact information for genetic counsellors specific to each eligible relative based on their geographic location. The National Society of Genetic Counsellors website (nsgc.org) was used both as a reference and to identify genetic counsellors near each of the eligible relatives.	3.2 Social support (practical)<br>Advise on, arrange, or provide practical help (e.g., from friends, relatives, colleagues, 'buddies' or staff) for performance of the behaviour	Theories of Learning; Goal-Setting Theory, Social Cognitive Theory
	personalized CD containing electronic versions of each resource	Not a behaviour change technique related to the behaviour of informing relative of their risk	-
	They were encouraged to disseminate these resources to relatives, and to contact their genetic counsellor for assistance and support throughout this process	1.3 Goal setting (outcome)<br>Set or agree on a goal defined in terms of a positive outcome of wanted behaviour.	Diffusion of Innovations Theory, Theories of Power, Models of Community Organisation, Self-Determination Theory, Goal-Setting

			Theory, Theories of Self-Regulation

Montgomery 2013

Time point	Intervention component	Behaviour change technique from V1 taxonomy	Relevant theory from Kok (2016) paper
Pre-disclosure session	Identifying relatives who could benefit from the information	1.3 Goal setting (outcome)<br>Set or agree on a goal defined in terms of a positive outcome of wanted behaviour.	Diffusion of Innovations Theory, Theories of Power, Models of Community Organisation, Self-Determination Theory, Goal-Setting Theory, Theories of Self-Regulation
	Choosing the communication format (phone, letter, email, etc.)	1.4 Action planning<br>Prompt detailed planning of performance of the behaviour (must include at least one of context, frequency, duration and<br>intensity).	Implementation intentions<br>(Theories of Goal Directed Behaviour)
	Assessing how much family members already knew and how much they might want to know	1.2 Problem solving<br>Analyse, or prompt the person to analyse, factors influencing the behaviour and generate or select strategies that include overcoming barriers and/or increasing facilitators.	Self-Determination Theory, Theories of Self-Regulation, Social Cognitive Theory, Attribution Theory; Theories of Goal Directed Behaviour.
Disclosure session	Sharing the actual genetic test result with family members	Not enough detail to assign BCT	-
	Responding to family members' emotional reaction to the disclosure	1.2 Problem solving<br>Analyse, or prompt the person to analyse, factors influencing the behaviour and	Self-Determination Theory, Theories of Self-Regulation, Social Cognitive Theory, Attribution Theory;

		generate or select strategies that include overcoming barriers and/or increasing facilitators.	Theories of Goal Directed Behaviour.
	Providing genetic counselling resources for family members	3.2 Social support (practical)<br>Advise on, arrange, or provide practical help (e.g., from friends, relatives, colleagues, 'buddies' or staff) for performance of the behaviour	Theories of Learning; Goal-Setting Theory, Social Cognitive Theory
	A resource guide outlining cancer risk factors, including family history, the benefits and limitations of genetic testing and the points covered in the six-step communication strategy was given to each intervention participant.	3.2 Social support (practical)<br>Advise on, arrange, or provide practical help (e.g., from friends, relatives, colleagues, 'buddies' or staff) for performance of the behaviour	See above

Eijzenga 2018 (MI)

Time point	Intervention component	Behaviour change technique from V1 taxonomy	Relevant theory from Kok (2016) paper
Phase 1	Exploring counselees' current and planned pattern of informing relatives	3.2 Social support (practical)<br>Advise on, arrange, or provide practical help (e.g., from friends, relatives, colleagues, 'buddies' or staff) for performance of the behaviour	Theories of Learning; Goal-Setting Theory, Social Cognitive Theory
	Systematically assessing knowledge about which relatives need to be	1.3 Goal setting (outcome)<br>Set or agree on a goal defined in	Diffusion of Innovations Theory, Theories of Power, Models of Community

	informed, and which information needs to be conveyed (using pedigree and summary letter)	terms of a positive outcome of wanted behaviour.	Organisation, Self-Determination Theory, Goal-Setting Theory, Theories of Self-Regulation
	Exploration of counselees' motives and (possible) resistance to inform relatives.	1.2 Problem solving<br>Analyse, or prompt the person to analyse, factors influencing the behaviour and generate or select strategies that include overcoming barriers and/or increasing facilitators.	Self-Determination Theory, Theories of Self-Regulation, Social Cognitive Theory, Attribution Theory; Theories of Goal Directed Behaviour.
Phase 2 (if participant has not informed all at-risk relatives correctly)	The "elicit-provide-elicit model"; first eliciting the person's understanding and information needs, then provide this information neutrally, followed by inviting the counselee to interpret the information.	3.2 Social support (practical)<br>Advise on, arrange, or provide practical help (e.g., from friends, relatives, colleagues, 'buddies' or staff) for performance of the behaviour	Theories of Learning; Goal-Setting Theory, Social Cognitive Theory
	Inviting the counselee to speak out arguments in favour of informing relatives to reinforce these arguments and thus strengthen the counselees' motivation.	3.2 Social support (practical)<br>Advise on, arrange, or provide practical help (e.g., from friends, relatives, colleagues, 'buddies' or staff) for performance of the behaviour	See above
	Inviting counselees' to discuss strategies they deem feasible to inform relatives.	1.2 Problem solving<br>Analyse, or prompt the person to analyse, factors influencing the behaviour and generate or select strategies that	Self-Determination Theory, Theories of Self-Regulation, Social Cognitive Theory, Attribution Theory; Theories of Goal Directed Behaviour.

		include overcoming barriers and/or increasing facilitators.	
	Brainstorm about possible solutions for their experienced barriers in informing at-risk relatives. To motivate and enlarge counselees self-efficacy, counselees are encouraged to list possible solution themselves.	1.4 Action planning Prompt detailed planning of performance of the behaviour (must include at least one of context, frequency, duration and intensity).	Implementation intentions (Theories of Goal Directed Behaviour)

1. Thomas BH, Ciliska D, Dobbins M, Micucci S. A process for systematically reviewing the literature: providing the research evidence for public health nursing interventions. Worldviews Evid Based Nurs. 2004;1(3):176–84.

2. Michie S, Richardson M, Johnston M, Abraham C, Francis J, Hardeman W, et al. The behavior change technique taxonomy (v1) of 93 hierarchically clustered techniques: building an international consensus for the reporting of behavior change interventions. Ann Behav Med [Internet]. 2013;46(1):81–95. Available from: http://www.ncbi.nlm.nih.gov/pubmed/23512568
